# Imaging hematomas in unforeseen sites - unveiling 2 cases of pediatric scurvy

**DOI:** 10.1186/s12887-026-06693-x

**Published:** 2026-03-05

**Authors:** Ting Ting Yew, Tjun Hoe Lui, Abdul Qayyum Abdul Rahman, Aun Nee Lim, Sue Lyn Tan

**Affiliations:** 1 Institute of Borneo Studies, Kota Samarahan, Sarawak Malaysia; 2 Department of Radiology, Faculty Medicine And Health Sciences, Universiti Malaysia Sarawak, Kota Samarahan, Sarawak Malaysia; 3 Department of Radiology, Sarawak General Hospital, Jalan Hospital, Kuching, Sarawak Malaysia; 4 Department of Pediatrics, Faculty Medicine And Health Sciences, Universiti Malaysia Sarawak, Kota Samarahan, Sarawak Malaysia

**Keywords:** scurvy, vitamin C deficiency, subperiosteal hematoma, proptosis, children

## Abstract

**Background:**

Scurvy, a nutritional disorder resulting from chronic deficiency in vitamin C, is now a rare occurrence in contemporary times. Nevertheless, instances can still arise in pediatric patients, typically linked to inadequate nutrition and socioeconomic factors. Diagnosing scurvy proves challenging due to its infrequency and nonspecific symptoms. Clinical and radiographic findings, coupled with low serum vitamin C levels, are crucial for diagnosis. Pediatric scurvy often exhibits prominent musculoskeletal manifestations with varying presentations.

**Case presentation:**

We present two cases of pediatric scurvy, wherein subperiosteal hematomas were identified on MRI, initially misdiagnosed as infections and abscesses. Both patients responded positively to vitamin C supplementation, and in one case, resolution of the hematoma was observed on MR imaging.

**Conclusions:**

Musculoskeletal manifestations, particularly subperiosteal hematomas, serve as significant indicators for diagnosing pediatric scurvy. Timely identification is crucial, as proper treatment yields an excellent prognosis.

## Background

Scurvy is generally rare nowadays; however, it is still found predominantly within specific vulnerable populations, particularly among the pediatric or elderly population with poor nutrition, individuals with neurodevelopmental disabilities or psychiatric conditions, and those with unique dietary habits or behavioral disorders that challenge their eating habits [1–3]. Diagnosing scurvy poses a challenge given its infrequency and nonspecific symptoms. Scurvy can manifest as subperiosteal hemorrhage, which can mimic osteoarticular infection, especially in a non-trauma setting. We report 2 cases of pediatric scurvy with subperiosteal hematomas evident on MRI, which were initially misdiagnosed as infections and abscesses.

## Case presentation

### Case 1

**Fig. 1:**
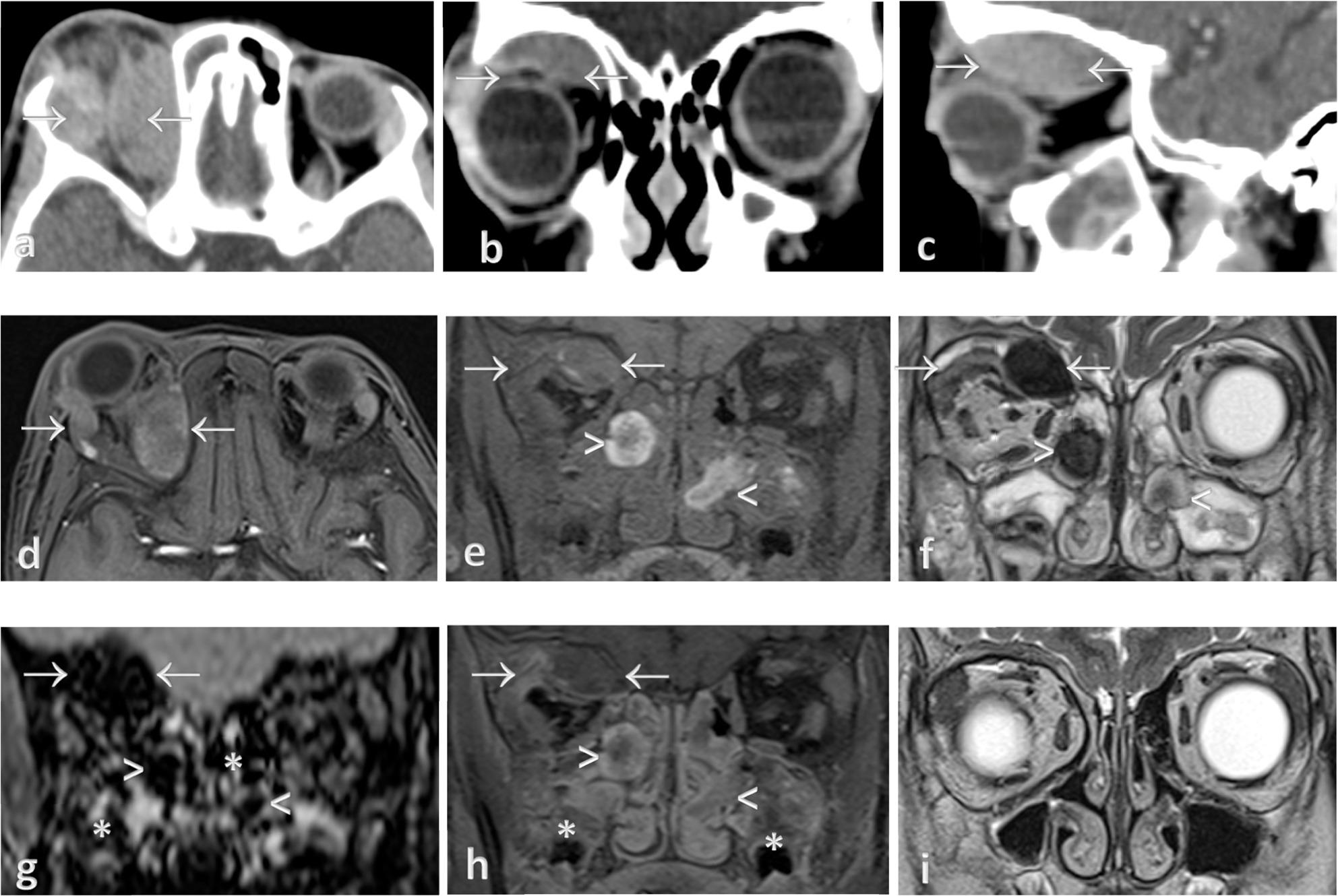
CT and MRI orbit of case 1. **a**, **b**, **c** Contrast-enhanced CT Orbit in axial, coronal, and sagittal images show rim-enhancing collections (arrow) at superior aspect of right orbital roof, extending to the medial and lateral orbital walls. **d**,** e**, **f** T1 fat -suppressed axial and coronal, T2w coronal images of the orbit show T1w isointense and mixed T2w hypointense to hyperintense collections at right orbital roof (arrow), with T1w heterogeneously hyperintense and T2w hypointense collections at bilateral nasal meati (arrow head). **g** SWI coronal image shows blooming artifacts at right orbital (arrow) and bilateral nasal meati (arrow head), and bilateral maxillary sinuses (asterisk). **h** T1 fat-suppressed post-contrast coronal image shows rim-enhancing collection at right orbit (arrow) with heterogeneously enhancing intranasal (arrow head) and intrasinal regions (asterisk). **i** T2w coronal image (6 months later) shows resolution of the collections

A 7-year-old girl with learning disability presented with gradual, painless swelling in her right eye for three days. There was no trauma or fever. Fundoscopy did not reveal any significant findings, and orbital cellulitis was suggested as the initial clinical diagnosis. However, a contrast-enhanced CT scan of the orbit displayed rim-enhancing collections along the superior aspect of the right orbital roof, extending to the medial and lateral orbital walls (Fig. 1a-c). No irregularities were observed in the globes or rectus muscles. She was treated for orbital cellulitis with periosteal collection and subsequently underwent right endoscopic transnasal orbital decompression and bilateral functional endoscopic sinus surgery (FESS), which blood clots were removed.

A subsequent MRI scan of the orbit showed hematomas of various ages with blooming artifacts (Fig. 1d-i) in the extraconal space of the right superior orbit, nasal meati, and bilateral maxillary sinuses. The patient had severe gum bleeding during intubation for the MRI under general anesthesia and also had a history of easy gum bleeding during dental visits previously. Further inquiry revealed that the lack of vegetables and fruits in her diet habits raised the suspicion of vitamin C deficiency. Her serum vitamin C was undetectable, thus she was started on high-dose vitamin C therapy, 300 mg four times daily for a week. Another FESS was performed to remove the residual blood clots to prevent secondary orbital infection. She was discharged with tablet vitamin C 300 mg daily for 3 months, albeit with some remaining right eye proptosis. During follow-up in the clinic, she was symptom-free and proptosis was resolved. A repeat MRI revealed complete resolution of the hematoma.

### Case 2

**Fig. 2:**
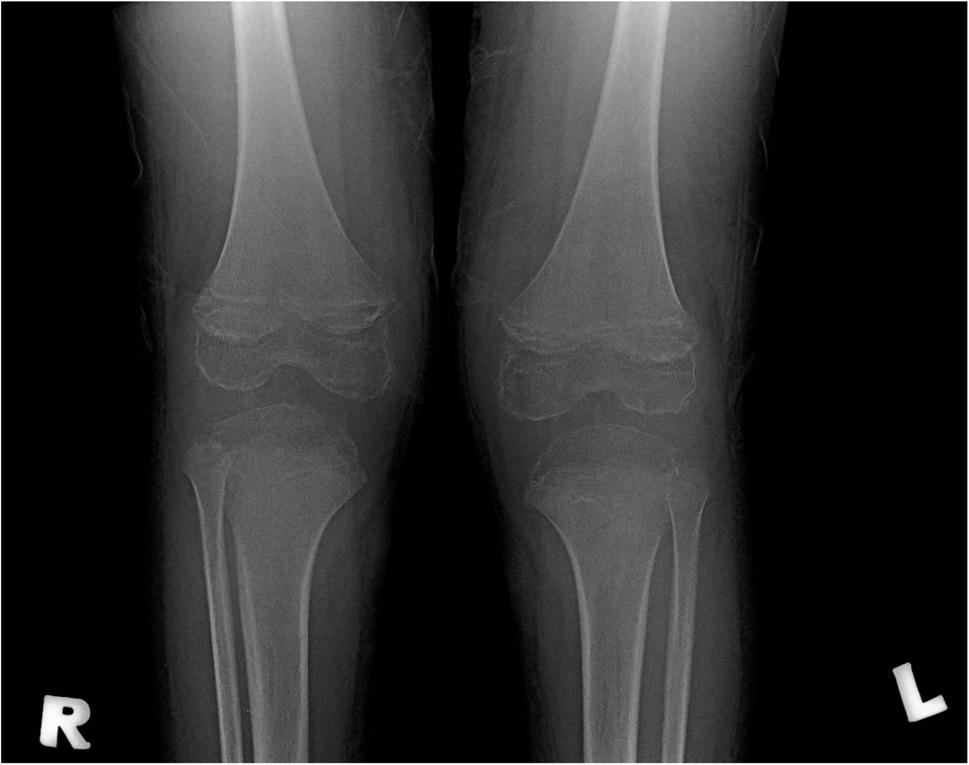
Bilateral knees radiographs show osteopenia and epiphyseal enlargement of bilateral femurs and tibias

A 2-year and 5-month-old boy with low socioeconomic status presented with sudden onset of bilateral lower limb pain and refused to ambulate for two days. No trauma or fever. He had right thigh and knee swelling with reduced range of movement. A blood workup excluded infection and blood dyscrasia. Bilateral knees radiograph showed osteopenia and enlargement of the epiphyses of the femur and tibia (Fig. 2).

**Fig. 3:**
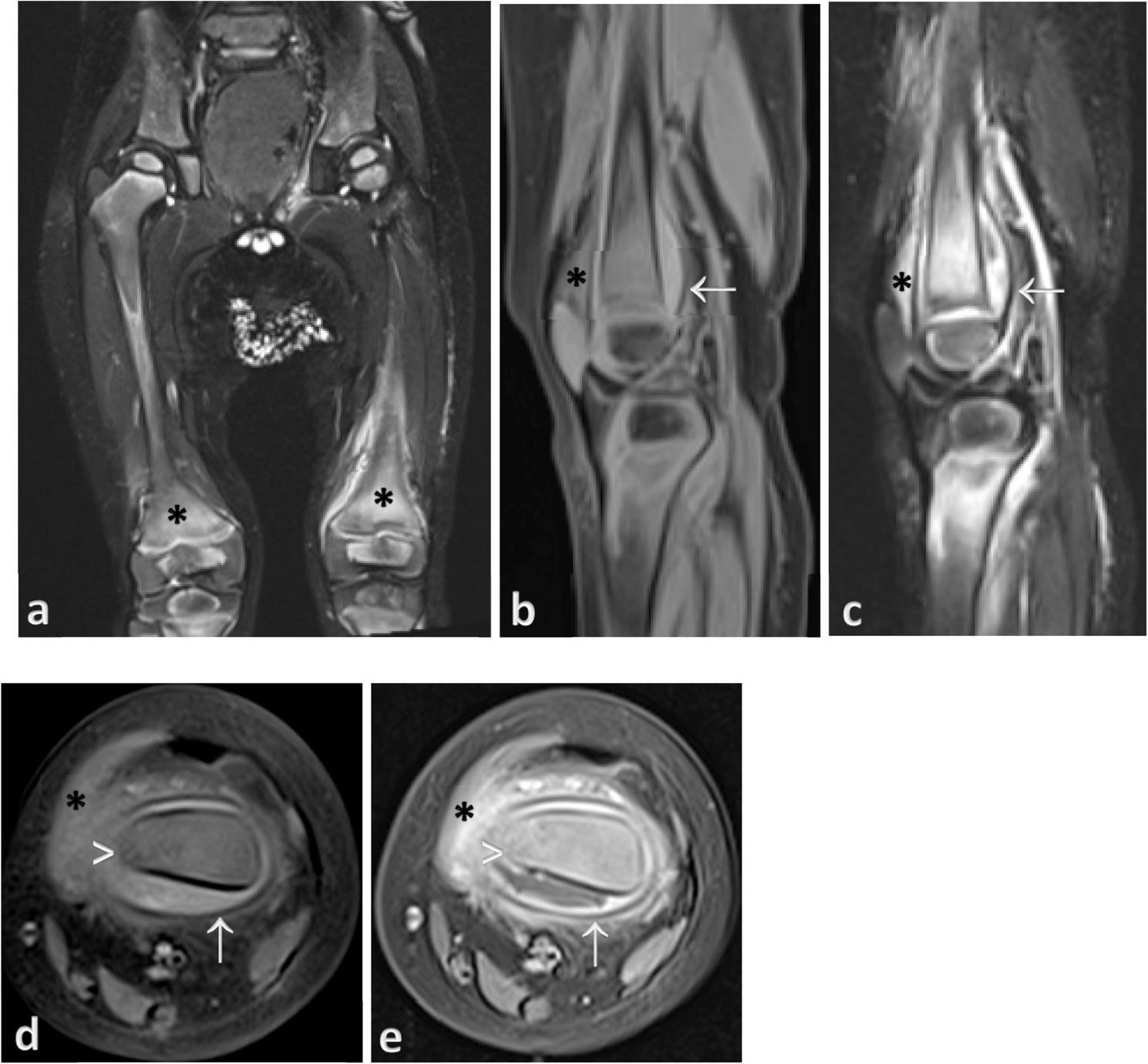
**a** T2 fat-suppressed coronal image shows diffuse marrow high signal changes (asterisk) within bilateral distal femurs and surrounding soft tissues. **b**, **c** T1 and T2 fat-suppressed sagittal images show high signal subperiosteal collection at left distal femur (arrow). There is also suprapatellar effusion (asterisk). **d**, **e** T1 fat-suppressed pre- and post-contrast axial images show rim-enhancing subperiosteal collection (arrow) at the left distal femur with enhancement of soft tissue superficial to periosteum (asterisk). Periosteal reaction is noted at the medial aspect (arrow head)

Further evaluation with MRI showed diffuse high marrow signals in bilateral femurs (Fig. 3a) with subperiosteal collections and knee effusions (Fig. 3b-e), the imaging differentials considered were osteomyelitis, abscesses, and septic arthritis. He was then started on intravenous Cloxacillin and Ceftazidine for the concern of osteoarticular infection despite no fever or leucocytosis. However, the symptoms persisted despite antibiotics. Left knee aspiration showed clear synovial fluid with a negative culture. In further history, he had gingivitis and poor gum health. Hence, scurvy was suspected. His low serum vitamin C (< 0.2 mg/dl) confirmed the diagnosis of scurvy, and a vitamin C supplement of 100 mg three times daily was started. During clinic review after two weeks, he was pain-free and able to walk with minimal support.

## Discussion

The clinical signs and symptoms of scurvy may appear within 4 to 12 weeks of inadequate vitamin C intake [4]. While most cases exhibit musculoskeletal symptoms like arthralgia, myalgia, hemarthrosis, muscle hemorrhage, and subperiosteal hematoma [5], hematological and oral manifestations like gum bleeding and anemia, or uncommon ocular manifestations such as subconjunctival, lid, anterior chamber, and retinal hemorrhages, along with proptosis [4, 6]. In some cases of unilateral proptosis, clinicians might initially consider an orbital infection or tumor rather than a hematoma caused by scurvy, leading to incorrect diagnosis, as observed in our case.

Typical cases of scurvy exhibit characteristic findings on plain radiographs of the immature skeleton, including diffuse demineralization and subperiosteal hemorrhages [7, 8]. These hemorrhages elevate the periosteum and prompt periosteal calcifications. Additional classic features involve epiphyseal rim scleroses linked to disorganized bone development in the center of ossification (Wimberger’s ring), a concentrated zone of provisional calcification in the metaphysis (white line of Frankel line), and metaphyseal spurs (Pelkin’s spur) causing metaphyseal cupping and potentially leading to corner fractures [8]. However, these radiographic features did not manifest in both of our patients.

Musculoskeletal manifestations are common in 80% of pediatric scurvy cases, which include subperiosteal hemorrhage [3, 9]. Proptosis due to orbital subperiosteal hematoma in children is uncommon and has been described in case reports [6, 9]. Orbital subperiosteal hematomas, though not exclusive to scurvy, usually occur in superior and subperiosteal regions; they can also result from facial trauma, barotrauma, Valsalva maneuver, bleeding disorders, anticoagulation therapy, or other systemic diseases [10]. For case 1, the patient presented with worsening proptosis of a short duration and was afebrile; therefore, orbital infection and malignancy are less likely to be considered. On CT, a subperiosteal hematoma appears as an internal hyperdense collection, whereas a subperiosteal abscess usually has a rim-enhancing wall with hypodense content. Given this distinction, reviewing the imaging retrospectively suggests that a subperiosteal hematoma should be considered in this patient, with a possible alternative diagnosis of subperiosteal collection with proteinaceous content, though this is less common. This was further supported by the MRI findings, where the hematoma collection showed T2 hypointensity, while an abscess would typically exhibits T2 hyperintensity. The T1 isointensity could be due to the age of the hematoma, as its appearance changes over time.

MRI is valuable in atypical cases of scurvy where the typical radiographic findings are absent. MRI findings associated with scurvy have been scarcely documented, typically exhibiting areas of bone marrow edema (decreased T1w and increased T2w signal intensity) predominantly within the metaphyses, subperiosteal fluid collection, or hematomas in the periosteum along the long bone shafts, with adjacent soft tissue signal abnormalities [3, 5, 11, 12]. While these imaging findings are non-specific and can also be observed in conditions like osteomyelitis, marrow disorders (anemia), or bone marrow replacement issues such as leukemia. In our cases, typical radiographic findings were absent, except for the orbital subperiosteal hematoma and bone marrow changes noted in the MRI. However, the hematomas in the medullary cavity and beneath the periosteum usually demonstrate heterogenous signals and higher T1 signal of blood compared to subperiosteal abscesses of osteomyelitis, along with the absence of cortical thickening or erosions, which highlight the important clues for diagnosing scurvy. For case 2, a T1 hyperintense subperiosteal collection suggests the possibility of a hematoma, as blood degradation changes its MR signal appearance with time. A study showed the greater amount and repeated occurrence of subperiosteal hematoma were crucial indicators for the diagnosis of scurvy, as subperiosteal hematoma were uncommon in other diseases [13]. Moreover, osteomyelitis or malignancy does not manifest oral and dermatological symptoms like gingival hematoma. As the diagnosis of osteoarticular infection shall be made on the basis of clinical findings and infective markers and positive bacteria culture with favorable response after empiric antibiotic therapy.

The diagnosis of scurvy is mainly based on medical history, dietary history of poor vitamin C intake, clinical assessment, radiological findings, and a low serum vitamin C level (< 0.2 mg/dl) [4]. Early recognition of the diagnosis prompts the initiation of empiric treatment with high-dose ascorbic acid. The best reliable evidence for diagnosing scurvy is the improvement of symptoms and resolution of the disease manifestations following vitamin C supplementation [3, 9]. The recommended dose is up to 300 mg per day for children to restore the diminished vitamin C levels, and the replacement typically occurs within 1 to 3 months or when all the signs and symptoms are resolved [4]. This was evident in both patients, who responded well to vitamin C treatment, with resolution of the hematoma observed in case 1 on follow-up MRI.

## Conclusions

Our cases shed light on the diverse and atypical presentations of scurvy, especially in the pediatric population. We highlight the importance of taking dietary history, it underscores the significance of comprehensive evaluation when atypical symptoms like orbital hemorrhage or subperiosteal hematoma arise.

Scurvy can be difficult to differentiate from infection on MRI in the absence of characteristic radiographic findings. Therefore, the presence of subperiosteal hematoma is an important clue to diagnose scurvy. A hyperdense subperiosteal collection on CT, along with T1 hyperintense and T2 hypointense appearance on MRI, should suggest a subperiosteal hematoma as the provisional diagnosis, with scurvy being the leading cause. Radiological imaging complemented with good clinical judgement and a low serum vitamin C level establishes the diagnosis. Early detection of scurvy is important to avoid unnecessary procedures or surgery and to prompt proper treatment for an excellent prognosis.

## Data Availability

The datasets used and analysed during the current study are available from the corresponding author on reasonable request.

## Abbreviations

CT: Computed tomography
MRI: Magnetic resonance imaging
FESS: Functional endoscopic sinus surgery
